# Vitamin D3/VDR resists diet-induced obesity by modulating UCP3 expression in muscles

**DOI:** 10.1186/s12929-016-0271-2

**Published:** 2016-07-29

**Authors:** Yue Fan, Kumi Futawaka, Rie Koyama, Yuki Fukuda, Misa Hayashi, Miyuki Imamoto, Takashi Miyawaki, Masato Kasahara, Tetsuya Tagami, Kenji Moriyama

**Affiliations:** 1 Department of Medicine & Clinical Science, Faculty of Pharmaceutical Sciences, Mukogawa Women’s University, Hyogo, 663-8179 Japan; 2 Department of Nephrology and Blood Purification, Institute of Biomedical Research and Innovation, Kobe Medical Frontier Center, Kobe, 650-0047 Japan; 3 Department of Food and Nutrition, Kyoto Women’s University, Kyoto, 605-8501 Japan; 4 Clinical Research Institute for Endocrine and Metabolic Diseases, National Hospital Organization Kyoto Medical Center, Kyoto, 612-8555 Japan; 5 School of Chinese Materia Medica, Beijing University of Chinese Medicine, Beijing, 100102 China

**Keywords:** VDRE, Transcription, Promoter

## Abstract

**Background:**

The impact of vitamin D3 (VD3) on obesity has been reported in the past. Our study was aimed at investigating the possible mechanisms by which VD3 affects obesity induced by a high fat diet.

**Methods:**

Eight-week-old C57BL/6 J male mice were fed a normal- or high-fat diet for 9 weeks and were treated with a gavage of vehicle (corn oil) or cholecalciferol (50 μg/kg, daily). Body weight, white adipose tissue weight, blood lipid and glucose levels were measured. In addition, we investigated the expression of 1,25(OH)_2_D_3_ (calcitriol)/VDR-regulated genes involved in energy and lipid metabolism, such as of uncoupling protein 3 (UCP3), by using qRT-PCR in the liver, adipose tissue, skeletal muscle and C2C12, L6, and H-EMC-SS cells. We also measured UCP3 promoter transcription in the same cell lines using a Dual Luciferase Assay. Furthermore, we analyzed the binding site consensus sequences of VDR on the UCP3 promoter.

**Results:**

Mice consuming a high-fat diet treated with cholecalciferol had lower body weight and adipose tissue weight and higher expression of UCP3 compared to the other treatment groups. Changes in the expression of genes correlated with calcitriol/VDR. Luciferase activity was dose-dependently associated with calcitriol/VDR levels. We confirmed the functional VDR binding site consensus sequences at -2200, -1561, -634, and +314 bp in the UCP3 promoter region.

**Conclusion:**

We suggest that VD3/VDR inhibits weight gain by activating UCP3 in the muscles.

**Electronic supplementary material:**

The online version of this article (doi:10.1186/s12929-016-0271-2) contains supplementary material, which is available to authorized users.

## Background

Obesity is associated with vitamin D deficiency, and both are areas of active public health concern. Vitamin D (1,25(OH)_2_D_3_) is a steroid hormone that has a range of physiological functions in skeletal and nonskeletal tissues, and can contribute to prevent and/or treat several diseases. We hypothesize that increased UCP3 expression promotes mitochondria activity and fatty acid oxidation. We provide evidence that VD3/VDR regulates body weight and white adipose tissues weight without changing food intake and blood lipid levels. We confirmed UCP3 gene transcription is initiated by 1,25(OH)_2_D_3_ (calcitriol)/VDR binding to the UCP3 promoter on the molecular basis, which reinforces energy metabolism, resulting in resistance to obesity caused by a high fat diet, as dealt with in this paper.

### Introduction

Obesity, most commonly caused by excessive dietary calories due to a high-fat diet, is increasing in prevalence worldwide [1]. Obesity is associated with several chronic diseases, including fatty liver, heart disease, type 2 diabetes mellitus, certain types of cancer, and osteoarthritis [2, 3]. The balance between energy intake and energy utilization maintains body weight. Energy is obtained by consuming calories and can be stored in adipose tissues or can be utilized by the body to sustain basic cellular functions and physical activities [4]. Since energy homeostasis is maintained by signals from feedback loops that regulate food intake, energy expenditure, lipid metabolism, and glucose metabolism [5], variation in the genes involved in these pathways likely influences the development of obesity.

Uncoupling proteins (UCPs) belong to the family of mitochondrial transporter proteins that are important in energy homeostasis. UCPs have become prominent in the fields of thermogenesis, obesity, diabetes, and free-radical biology and have been considered candidate genes for obesity and insulin resistance [6]. Among the five subtypes of UCPs, UCP3 is a member of the mitochondrial transporter super family and is expressed predominantly in skeletal muscles [7, 8]. UCP3 mediates energy expenditure via mitochondrial proton leak and lipid metabolism [9–12] and is involved in fatty acid translocation [13]. Reduction in the function or expression of UCP3 likely decreases energy expenditure or increases fat storage of energy [14]. UCP3 is negatively correlated with body mass index (BMI) [15], suggesting a potential link between UCP3 and obesity, potentially by the proposed roles of UCP3 in facilitating fatty acid oxidation [16] and preventing triglyceride accumulation [17]. Therefore, UCP3 likely modulates body weight, but further investigation is needed to confirm this.

Vitamin D (VD) is a fat-soluble vitamin that is either synthesized in the skin after exposure to solar UVB radiation or obtained from the diet. VD regulates both bone metabolism and calcium-phosphorus homeostasis [18]. In humans, vitamin D3 (VD3, cholecalciferol) is transformed to 1, 25-dihydroxyvitamin D_3_ (1,25(OH)_2_D_3_) (calcitriol), the most active form [19]. Calcitriol functions are primarily activated by its nuclear receptor, vitamin D receptor (VDR), which recruits its preferred dimerization partner, retinoid X receptor (RXR), to form a heterodimer. This complex acts as a ligand-dependent transcription factor that combines with vitamin D response elements (VDRE) in the promoter regions of target genes [20, 21].

Recent epidemiological investigations found that low serum calcitriol levels are associated with a higher BMI and greater body fat [22, 23]. Additionally, elevated dietary VD intake and serum 25(OH)D levels are associated with reduced omental adipocyte size and lower visceral adiposity, which have important clinical implications [24]. Increasing evidence suggests that VD3 supplementation limits weight gain induced by high-fat diets due to increased lipid oxidation [25]. Administration of calcitriol regulates peroxisome proliferator-activated receptor α (PPARα), which prevents high-fat diets-induced body weight gain by inhibiting lipogenesis [26]. Reports regarding VD3 and obesity have been contradictory; for example, VDR knock-out mice are resistant to high-fat diets-induced weight gain [27] and show a slower growth rate and accumulate less fat mass [28]. Therefore, further research on the effect of VD3/VDR on obesity is needed. To investigate the influence of VD3/VDR on body weight and lipid, we investigated the energy metabolism in obese mice consuming a high-fat diet and a possible consensus sequences in the UCP3 promoter for VDR.

## Methods

### Animal and treatment

Eight-week-old male C57BL/6 J mice (Japan SLC, Shizuoka, Japan) were acclimated in the animal facility under a 12-h light/dark cycle prior to the experiment. For 9 weeks, the mice were fed a control normal-fat diet (group NFD: 10 kcal% fat (lard)), (D12450B, Research Diets Inc, NJ, US) or a high-fat diet (groups HFD and HFVD: 45 kcal% fat (lard)), (D12451, Research Diets Inc). We used HFD feeding as previously described [25]. Both NFD and HFD mice were treated with vehicle (1 % ethanol, 9 % pure water, 90 % corn oil). HFVD mice were treated with 50 μg/kg body wt/d cholecalciferol (VD3) (C0314, TCI, Tokyo Japan) administered by gavage daily. We determined the dose of VD3 to feed as previously described [18]. Body weight was measured every day and food intake was calculated per cage. Mice were fasted 24 h before sacrificing, at which point perfused normal saline from the left ventricle of the heart, blood, liver, mesenteric adipose, kidney adipose, epididymis adipose, and thigh rectus femoris muscle were collected and analyzed.

### Blood serum analysis

Mouse blood samples were placed on ice for 1 h and then centrifuged at 3500 × g for 90 s. The blood serum was collected and total cholesterol (T-CHO), low density lipoprotein-cholesterol (LDL-C), high density lipoprotein-cholesterol (HDL-C), and triglyceride (TG) were measured using LabAssay™ Cholesterol (294-65801, Wako Pure Chemical Industries, Ltd, Japan), LabAssay™ Glucose (298-65701), LabAssay™ Triglyceride (290-63701), according to manufacturer’s instructions.

### Tissue sample RNA extraction for quantitative real-time PCR and PCR agarose electrophoresis

Total RNA was extracted from homogenized ~1 mm sliced tissue samples using the RiboZol RNA extraction reagents (N580, AMRESCO, US). cDNA was synthesized using iScript Reverse Transcription Supermix (170-8841, BIO-RAD, US) and the following conditions: 25 °C for 5 min, 42 °C for 30 min, 85 °C for 5 min, 4 °C until use. Real-time qPCR was conducted using SsoAdvanced Universal SYBR Green Supermix (172-5271,BIO-RAD). The cycle conditions were as follows: 95 °C for 1 min followed by 39 cycles of 95 °C for 5 s and 59.5 °C for 10 s. The level of UCP3 mRNA was evaluated from thigh rectus femoralis muscle samples and the housekeeping gene GAPDH was used as an internal control. Expression of liver X receptors (LXRs), farnesoid X receptor (FXR), peroxisome proliferator-activated receptor (PPAR), and related genes were measured in the same methods.

To amplify VDR and β-actin, PCR was performed as follows: 30 cycles of 98 °C 10 s, 55 °C 30 s, and 72 °C 15 s. VDR and β-actin PCR products (188 bp and 192 bp, respectively) were confirmed by 2 % agarose gel electrophoresis, ethidium bromide staining, and UV light visualization. The primers used are listed in Additional file 1: Table S1.

### Cell culture

Mouse C2C12 muscle cells (C2C12), rat L6 muscle cells (L6), H-EMC-SS chondrosarcoma cells, and human embryonic kidney cells (HEK293) were purchased from JCRB Cell Bank (National Institute of Biomedical Innovation, Tokyo, Japan). Cells were maintained in Dulbecco’s modified Eagle’s medium (DMEM) (05919, Nissui Pharmaceutical, Japan) containing 10 % FBS (SH30910.03, Thermo Scientific, US), 1 % penicillin (168-23191, Wako) and 1 % L(+) - Glutamine (074-00522, Wako) at 37 °C in 5 % CO2. Cultures were passaged in a subcultivation ratio of 1:3 - 1:4 every two days using 0.25 % w/v Trypsin-1 mmol/L EDTA-4Na Solution (201-16945, Wako).

### Plasmid construction

The human UCP3 promoter was generously gifted by Dr. Jean A. Boutin (Biotechnologie, Pharmacologie Moléculaire et Cellulaire, Institut de Recherches SERVIER, France). The UCP3 promoter was digested with XbaI and NcoI and inserted into the pGL4.10-basic vector (Promega Corp., WI) containing the luciferase gene (luc), resulting in a luciferase eukaryotic expression plasmid, which we named UCP3-pro-Luc. The plasmid for mouse UCP3 mRNA interference was generously gifted by Prof. Wolfgang F. Graier (Institute of Molecular Biology & Biochemistry, Center of Molecular Medicine Medical University of Graz, Austria).

### Cell transfection, calcitriol stimulation, and quantitative real-time PCR (qRT-PCR)

We measured mRNA expression in C2C12, L6, and H-EMC-SS untransfected cells or cells transfected with pCMX-VDR, expression plasmid of VDR (pCMX-VDR was provided by K. Umesono and R. M. Evans (Salk Institute, San Diego, CA)). One dish (100 mm) of 5–6 X 10^5^ cells were seeded in a 6-well plate and changed to Opti-MEM (31985-070, Gibco, US) until the cells were 70–80 % confluent. pCMX-VDR (100 ng/well) was transfected using Lipofectamine 2000 DNA transfection reagent (11668-019, Invitrogen, Life Technologies, US) for 5 h then the medium was changed to DMEM. Calcitriol (50 nM) was added and cells were incubated for 24 h. For mRNA analysis, total RNA was isolated using the RiboZol RNA extraction reagents. cDNA synthesis and real time PCR were conducted as described above. UCP3 mRNA expression was measured in C2C12, L6, and H-EMC-SS cells. The housekeeping gene GAPDH was used as an internal control.

### Calcium phosphate cell transfection, calcitriol stimulation, and dual luciferase assay

The reporter plasmid (UCP3-pro-luc, 100 ng/mL), expression plasmid of VDR (pCMX-VDR, 50 ng/mL), and internal control plasmid (pGL 4.70 10 ng/mL) were transfected into HEK 293, C2C12, and H-EMC-SS cells using 0.01 M Tris-HCl, 2.5 M CaCl2, and 2 × HBS. About 5 h after transfection, the media was changed to DMEM. A gradient of calcitriol (0.1–100 nM) was added and the cells were stimulated for 24 h. The cells were harvested using 1× Passive Lysis Buffer (E194A, Promega, WI) and measured by the Dual Luciferase Reporter Assay System (E1960, Promega, Corp. WI) according to the manufacturer’s instructions.

### Candidate VDRE sequence search using the UCP3 promoter deletion model

PSORTII (PSORTII computer software provided by the Human Genome Center, Institute for Medical Science, Tokyo University, Japan) was used to detect putative VDREs. All settings used were default settings. Of the detected sequences, those with significant similarities or identical to the mouse osteopontin were selected [29]. Then we generated five deletion constructs containing the candidate VDREs to screen for functional regions (Fig. 5a). Next we subcloned ten putative VDREs from the 5’-flanking region of UCP3 and prepared ten reporter constructs, each containing a VRDE candidate as previously prescribed [30]. In briefly, the reporter plasmid VDRE-tk-luc contains one copy of the mouse osteopontin VDRE upstream of a 151 bp truncated thymidine kinase promoter, inserted between the SacI and HindIII sites of the pGL 4.10-basic vector and was used as the positive control. Each candidate was inserted into VDRE-tk-luc substituting for the mouse osteopotin VDRE. The candidates consisted of -2200: designates as ①, -2043: ②, -1867: ③, -1561: ④, -634: ⑤, -595: ⑥, -80: ⑦, -1: ⑧, +162: ⑨, and +314 bp :⑩ of the UCP3 promoter. The primers used for reporter plasmid construction are listed in Additional file 1: Table S2. The relative luciferase activity of each reporter was compared with the positive control using the protocol described above.

### Statistical analysis

The results are shown as the mean ± SD. The significance of differences was determined by one-way ANOVA and *post hoc* Bonferroni/Tukey test for multiple comparisons using SPSS 17.0 software. A p-value of *P* < 0.05 was considered statistically significance.

## Results

### VD3 supplementation protected against high fat diet-induced obesity

#### VD3 limited the body weight gain of HFD mice

**Fig. 1:**
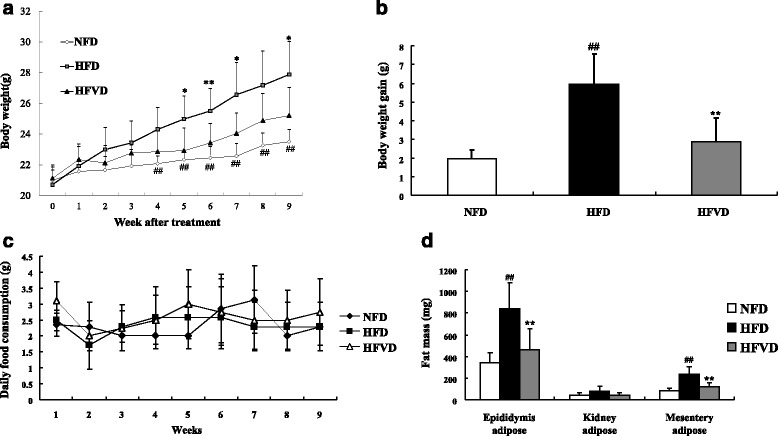
VD3 decreased body weight and fat mass of HFD group mice. The individual body weight (**a**) and the food consumption (**c**) were measured daily and weekly, respectively. After 9 weeks, body weight gain was analyzed among each group (**b**). VD3 altered fat mass of mice fed a high-fat diet (**d**). *n* = 7. Compared to NFD, ##; *P* < 0.01. Compared with HFD, *; *P* < 0.05, **; *P* < 0.01

High-fat diet feeding mice treated with cholecalciferol (HFVD) gained less weight compared to the HFD group (Additional file 2: Figure S1). After the fifth week, the difference in weight gain between groups was significant (Fig. 1a, *P* < 0.05). The weight gain of HFD mice was 206.7 % of that of the NFD group. On the other hand, the weight gain of HFVD mice was 51.8 % compared to the NFD group (Fig. 1b, *P* < 0.01). Food consumption was not different between each group (Fig. 1c).

#### VD3 decreased fat mass in HFD mice

We measured the tissue weight of epididymis adipose, kidney adipose, and mesentery adipose to investigate fat distribution. The weight of epididymis adipose and mesentery adipose was significantly higher in the HFD group compared to the NFD group by 142.8 and 184.0 %, respectively (*P* < 0.01). Treatment with cholecalciferol increased tissue weight by 44.4 and 48.4 %, respectively (*P* < 0.01) compared to NFD mice (Fig. 1d).

#### VD3 had no effects on altering serum lipid

Glucose and serum lipids include TC, LDL-C, HDL-C, and TG. The serum levels of the HFD and the HFVD groups had no significant change when compared with the NFD group (data not shown).

#### VD3 elevated the mRNA expression of UCP3 in the thigh rectus femoris muscle

**Fig. 2:**
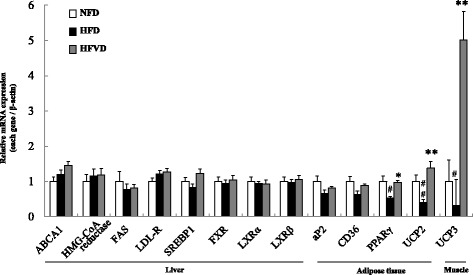
VD3 increased UCP3 mRNA expression in muscles. The effects of VD3 on UCP3 mRNA expression in the thigh rectus femoris muscle of mice fed a high-fat diet. *n* = 5. Compared with NFD, **; *P* < 0.01

To explore the potential mechanism of the reducing body weight effects of cholecalciferol, we examined the mRNA expression of various target genes that were reported to be involved in lipid, glucose and energy metabolisms, paying special attention to UCP genes. We measured UCP3 mRNA levels in the thigh muscle using qRT-PCR. The expression of UCP3 mRNA in the thigh muscle increased in the cholecalciferol treated HFVD group compared with the untreated HFD group (*P* < 0.01, Fig. 2a). We also examined that VDR was present in white adipose tissue, liver, and muscle by PCR agarose electrophoresis in every group (Additional file 3: Figure S2). These results suggest cholecalciferol decreases body weight and may alter energy metabolism by involving UCP3 (Fig. 2b). We also observed the expression level of mRNA of UCP1 in the brown and white adipose tissues. Those were not elevated by administration of VD3 (Additional file 4: Figure S3).

### Relative luciferase activities of UCP3-pro-luc were dose dependent on calcitriol in three muscle cell lines

**Fig. 3:**
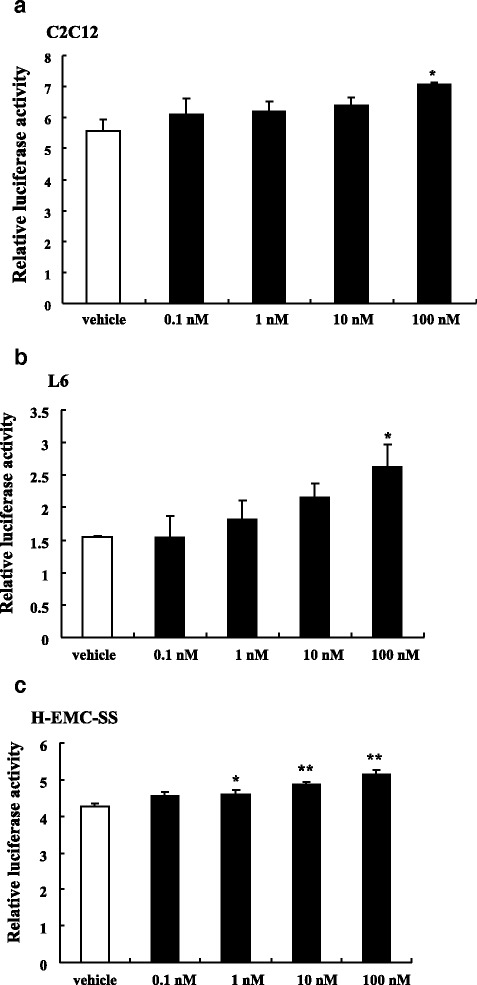
Calcitriol enhanced relative luciferase activity in a dose dependent manner. UCP3-pro-luc was transfected into C2C12 cells (**a**), L6 cells (**b**), and H-EMC-SS cells (**c**). Relative luciferase activity was analyzed by Dual Luciferase Assay after stimulation with calcitriol. *n* = 3, compared with vehicle, *: *P* < 0.05; **: *P* < 0.01

To determine the effects of calcitriol on relative luciferase activities of UCP3-pro-luc, we used the Dual Luciferase Assay in C2C12 cells (Fig. 3a), L6 (Fig. 3b), and H-EMC-SS (Fig. 3c). Compared with the vehicle control, luciferase activity was significantly increased in a dose dependent manner as concentrations of calcitriol (0.1–100 nM) increased. The UCP3-pro-luc promoter activity in H-EMC-SS cells was enhanced by 1 nM (*P* < 0.05), 10 nM (*P* < 0.01), and 100 nM (*P* < 0.01) calcitriol. Similar effects were observed in both L6 and C2C12 cells by 100 nM calcitriol (*P* < 0.05).

### UCP and nuclear receptor genes were affected by calcitriol especially combined with VDR

**Fig. 4:**
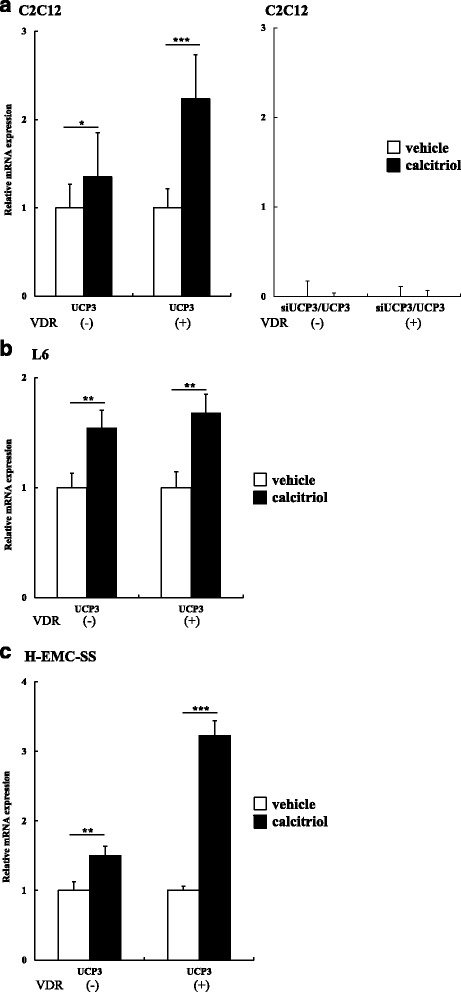
Calcitriol increased UCP3 mRNA expression in muscle cells. Relative mRNA expression was detected in C2C12 cells (**a**), L6 cells (**b**), and H-EMC-SS cells (**c**) using qRT-PCR. To investigate the role of calcitriol and VDR on UCP3 mRNA expression, we transfected with a control plasmid ((-) mock) or VDR expression plasmid ((+) pCMX-VDR). We also performed loss-of-function experiments using selective siRNA against UCP3 (**a**). Compared with non-transfected controls, siRNAs against UCP3 strongly reduced the expression of the UCP3. *n* = 3. Compared with vehicle, *; *P* < 0.05, **; *P* < 0.01. ***; *P* < 0.005

To detect the effects of VD3 on energy metabolism, we analyzed the expression of UCP3 mRNA in C2C12 (Fig. 4a), L6 (Fig. 4b), and H-EMC-SS (Fig. 4c) both untransfected and transfected with VDR. In untransfected VDR C2C12 cells, UCP3 mRNA increased in the presence of 50 nM calcitriol (*P* < 0.05). In VDR transfected cells, the same patterns were amplified (*P* < 0.01). The siRNAs against mouse UCP3 diminished expression of the respective transcript (Fig. 4a).

### Identification of a functional consensus sequence of VDR binding sites in the UCP3 promoter

**Fig. 5:**
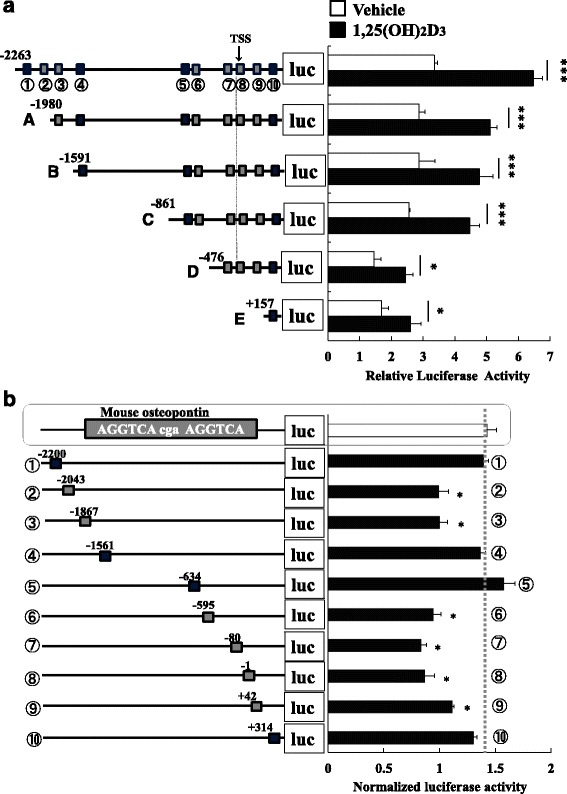
VD3 responsiveness of candidate VDRE upon transient transfection. The schematic structure of the proximal region of the human UCP3 promoter is shown; nucleotides are numbered relative to the transcriptional start site (TSS). The number below the top line denotes the position of the putative VDRE in the UCP3 promoter as follows; -2200; ①, -2043; ②, -1867; ③, -1561; ④, -634; ⑤, -595; ⑥, -80; ⑦, -1; ⑧, +162; ⑨, and +314; ⑩. Each truncated UCP3-pro-luc reporter construct and CMX-hVDR were co-transfected into HEK-293 cells and relative luciferase activity was compared. **a** Error bars represent SD from at least three experiments. *n* = 3. Compared with the control, *; *P* < 0.05, ***; *P* < 0.005. Solid boxes represent putative VDRE with high sequence similarity to the positive control (more than 80 %). Shadow boxes represent those with medium similarity (more than 70 %), open boxes represent those with relatively low similarity (more than 60 %) to the positive control. **b** As a positive control for VD3 responsiveness, we used the mouse osteopotin VDRE (AGGTCAcgaAGGTCA). Relative luciferase activity of each putative VDRE-luc-reporter construct compared to the positive control. Each value is expressed as mean ± SD from at least three experiments. Both designated nucleotide number relative to the TSS and sequential number is shown of the putative VDREs examined. *; *P* < 0.05 compared to the positive control

All truncated reporter constructs had at least some promoter activity although the actual strength was dependent on the length of UCP3 promoter (Fig. 5a). Above all, abrogation between -861 and -476 on the UCP3 promoter significantly decreased the transcriptional activity to 54.7 ± 2.4 % of that -861UCP3-pro-Luc (Fig. 5a). Therefore, the results indicate that there are multiple functional VDREs in the UCP3 promoter. We examined luciferase activity of our generated reporter constructs containing putative VDREs. The magnitude of activation was dependent on sequence similarity to the osteopontin VDRE (AGGTCAcgaAGGTCA) (Fig. 5b, Additional file 1: Table S2 and S4). These results suggest that the functional VDREs are located at -2200, -1561, -634, and +314 bp of the proximal region of the UCP3 promoter.

## Discussion

In this study, we determined the effects of VD3 on obesity in mice fed high-fat diets. Body weight, weight gain, and white adipose tissue decreased in mice treated with VD3. Some metabolism genes, including UCP3, had altered gene expression in VD3 treated mice, suggesting variation in mitochondrion function might be involved. In addition, we identified the functional consensus sequence of calcitriol/VDR in the UCP3 promoter.

Some researchers have shown that liganded VDR represses PPARγ expression via inhibition of C/EBPβ expression and inhibits adipogenesis [31, 32]. Other studies suggest that the anti-adipogenic effect of calcitriol-suppressing transcription factor PPARγ is mediated by WNT10B and nuclear β-catenin expression levels in 3 T3-L1 preadipocytes [33]. To examine if VD3 decreases obesity, we fed C57BL/6 mice a high-fat diet for 9 weeks to mimic the pathogenesis of obesity, which results in increased body weight gain and other metabolic disturbances by affecting food intake and peripheral metabolism [34]. Based on the previously described association between obesity and VD3, we investigated the role of VD3 on modulating body weight. Body weight significantly increased after 4 weeks for the HFD group while treatment with VD3 (HFVD) resulted in decreased body weight beginning the fifth week compared to the HFD group (Fig. 1a). Similar to changes in body weight, VD3 supplemented mice gained less weight than HFD mice in spite of that all mice have the almost same original body weight. However, daily food consumption was almost constant, implying appetite was not affected between groups and that differences in weight gain cannot be attributed to differences in calorie intake (Fig. 1c). We measured the mass of visceral adipose tissue and observed changes in the weight of epididymis adipose and mesentery adipose in HFD mice in accordance with changes in body weight (Fig. 1d). We speculate that decreased adipose tissue weight in VD3 supplemented mice is due to the inhibition of adipogenesis.

Levels of blood lipid total-cholesterol, LDL-cholesterol, HDL-cholesterol, triglyceride, and glucose were comparable between treatments, suggesting the variation in body weight and adipose tissue did not affect serum lipid levels (data not shown). To investigate the mechanism of VD3-associated inhibition of weight gain, we measured mRNA levels by qRT-PCR in muscle and adipose samples. The results show weight loss by VD3 supplementation may occur by UCP3 in thigh rectus femoris muscle, suggesting VD3 may influence energy metabolism and thereby alter body weight. Recent findings also highlight a new role for calcitriol and its receptor VDR in adipose tissue energy homeostasis [35], especially in regulating UCP expression [28]. We identified the existence of VDR by PCR gel electrophoresis, suggesting that VD3 modulates body weight by interacting with its receptor VDR.

To explore the role of VD3 in the modulation of body weight, we performed in vitro experiments using C2C12, L6, H-EMC-SS cells to observe the expression of genes involved in energy and lipid metabolism, including UCP3. These three cell types represent both different species and different muscle cell types. We employed H-EMC-SS cells, as substitutes for myocyte or myoblast cell lines to examine VD3 action on human derived cell line. We demonstrated that calcitriol upregulates the expression of UCP3 in all cell types, with even higher expression in pCMX-VDR transfected cells. Those results underline that UCP3 gene in muscle cell lines are positively regulated by VDR-mediated gene transcription. Furthermore, we verified the presence of VDR in these cells by examining the expression of VDR (Additional file 5: Figure S4). Expression levels of VDR mRNA were varied depending on the cell lines, though the level of them were less than that of the skeletal muscle. To verify the effect of calcitriol on relative luciferase activities, we used the Dual Luciferase Assay in UCP3-pro-luc transfected cells. The relative luciferase activities increased in response to calcitriol in a dose dependent manner in all cell lines. These data indicate that treatment of muscle cells with calcitriol increases the promoter activity in the presence of UCP3. Therefore, calcitriol enhances the expression of UCP3 in muscle cell lines via binding to VDR.

UCP and respiration uncoupling are implicated in numerous physiological and pathological processes, including adaptive thermogenesis [36], regulation of fatty acid oxidation [37], and body weight regulation [38]. UCP3 protects against lipid-induced mitochondrial damage by facilitating the export of fatty acids that cannot be oxidized from the mitochondrial matrix [39]. The previous report revealed that the moderate overexpression of UCP3 led to an increase in mitochondrial oxygen consumption and reduced oxidative phosphorylation efficiency in skeletal muscle fibers [40]. Overexpression of UCP3 mRNA in mouse skeletal muscle reduces diet-induced obesity, suggesting that UCP3 has therapeutic potential in the treatment of obesity [38]. UCP3 is thought to regulate mitochondrial fatty acid oxidation. For example, gastrocnemius muscle mitochondria isolated from UCP3^+/+^ mice can better oxidize fatty acids and result in lower fatty acid levels than those of UCP3^+/−^ and UCP3^−/−^ mice [41]. UCP3 protects against body weight and fat gain [42] and prevents accumulation of triglycerides in both adipose tissues and muscles [43] during high-fat feeding. Higher levels of fat are stored in mice lacking UCP3 that are fed high-fat diets [17]. In skeletal muscles, reduced mitochondrial uncoupling impairs mitochondrial fatty acid oxidation, leading to fatty acid and/or metabolite accumulation and increased oxidative stress, which results in obesity [44, 45].

We showed a significant increase in UCP3 mRNA expression by VD3 in thigh rectus femoris muscles. Additionally, UCP3 mRNA expression increased in both untransfected and pCMX-VDR-transfected C2C12, L6, and H-EMC-SS cells. The relative luciferase activity increased in a calcitriol-dose-dependent manner in three different muscle cells transfected with the UCP3-pro-luc vector. Therefore, we speculate that UCP3 facilitates mitochondrial fatty acid oxidation and decreases oxidative stress, thus slowing the progression of obesity.

Finally, we analyzed the UCP3 promoter and VDR for binding site consensus sequences. Deletion analysis of the UCP3 promoter constructs indicated that the VDREs are located at -2200, -1561, -634, and +314 bp. Particularly, without the No. 5 VDRE sited at -634, the UCP3 promoter activity of transcription decreased dramatically -45.3 ± 2.4 % in comparison to its control. So we deduced that the transcriptional activities of calcitriol/VDR primarily depend on its sequence similarity to that of osteopontin, which are known to the most potent VDRE to date. Our results indicate that at least four VDRE-binding site consensus sequences on the UCP3 promoter control the transcriptional regulation of UCP3 synergistically depending on the calcitriol/VDR and/or RXR conditions. The liganded VDR binds VDRE on the promoter of target genes and regulates their expression via the formation of heterodimeric complexes with RXR. The VDR also can interact with nuclear receptor, such as PPARs, thyroid hormone receptors, corepressors, coactivators, and many transcriptional factors [30, 46]. On the contrary, UCP3 gene promoter harbors several cis-elements essential for expression [47–49]. The relative contribution of those in the regulatory regions to UCP3 gene expression remains to be clarified.

UCPs are a highly useful molecular target for the treatment of obese patients for whom dietary restriction and exercise are difficult. However, because the signal transducers that function downstream of calcitriol/VDR also participate in high calcemia and urinary stones, investigations into the clinical applications of VD3 and/or calcitriol should be conducted cautiously. Therefore, drugs that selectively regulate VDR, which is expressed in myocytes, and intentionally regulate only UCP3 may be used as seeds for further drug development. Such drugs may be beneficial for supplementing weight reduction drugs and for obese patients.

## Conclusions

In summary, the present study demonstrates that the regulation energy metabolism, which was hypothesized to be directly acted on by 1,25(OH)_2_VD_3_, involves UCP3 via VDR. Furthermore, UCP3 and VDR is expressed widely throughout the skeletal muscle in the whole body. This suggests that circulating 1,25(OH)_2_VD_3_ are related to the direct effects of local thermogenesis and energy metabolism.

## Additional files

Additional file 1
**Table S1.** Primer sets used for qRT-PCR. **Table S2.** Primer sets used for plasmid construction and the VDRE promoter assay. **Table S3**. VD3 responsiveness of candidate VDRE upon transient transfection. (DOC 55 kb) Additional file 2: Figure S1. Comparison of mesenteric fat tissue of representative mouse originated from the breeding stock of each group. Similar phenomena were noticed at the epididymis adipose tissues. (PPTX 281 kb) Additional file 3: Figure S2. VDR mRNA expression was detected in the (1) kidney adipose, (2) mesentery adipose, (3) epididymis adipose, (4) liver and (5) thigh rectus femoralis muscle via PCR gel electrophoresis. The sequences of the primer sets are shown in Additional file 1: Table S1. PCR products of the expected sizes were detected by agarose gel electrophoresis, suggesting that VDR transcripts were present in those tissues. (PPTX 88 kb) Additional file 4: Figure S3. UCP1 mRNA expression was detected in the abdominal and epididymal adipose via quantitative real time PCR. The sequences of the primer sets are shown in Additional file 1: Table S1. The expression level of UCP1 in the abdominal adipose tissues of HFDV were less than that of HFD. On the contrary, there was no significant difference between HFD and HFVD in expression level of UCP1 in the epididymal adipose tissue. UCP1 mRNA levels seem to be inconstant, depending on the relative content of browning cell number per volume of the fat. Although HFD treatment had modified the UCP1 mRNA expression levels even in the white adipose tissues, VD3 administration did not have any particular contributions to UCP1 expression to resist diet-induced obesity. (PPTX 47 kb) Additional file 5: Figure S4. The presence of VDR transcripts was confirmed by RT-PCR analysis of C2C12 cells, L6 cells, H-EMC-SS cells and the rectus femoris muscle. PCR products of the expected sizes were detected by agarose gel electrophoresis, suggesting that the transcripts were present in each cell line. Beta actin was used as a positive control for PCRs. (PPTX 49 kb)
