# Supplementary material for: Vitamin D3/VDR resists diet-induced obesity by modulating UCP3 expression in muscles
Source: J Biomed Sci. 2016 Jul 29;23:56. doi: 10.1186/s12929-016-0271-2 (PMC4966724; doi:10.1186/s12929-016-0271-2)
Supplement: Additional file 1 — Table S1. Primer sets used for qRT-PCR. Table S2. Primer sets used for plasmid construction and the VDRE promoter assay. Table S3. VD3 responsiveness of candidate VDRE upon transient transfection. (DOC 55 kb) [file 12929_2016_271_MOESM1_ESM.doc]

**Additional file 1 (Table S1-S3)**

**Table S1.** Primer sets used for qRT-PCR

| Gene | Forward primer | Reverse primer |
| --- | --- | --- |
| GAPDH | 5’-TGTCTGTCTTTGTCCTTGAGAGG-3’ | 5’-GTCTTGGTGGCCCAGGTT-3’ |
| UCP3 | 5’-TACCCAACCTTGGCTAGACG-3’ | 5’-GTCCGAGGAGAGAGCTTGC-3’ |
| UCP1 | 5’-GTGAAGGTCAGAATGCAAGC | 5’-AGGGCCCCCTTCATGAGGTC |
| LXRα | 5’-GCATCACCTTCCTCAAGGACT-3’ | 5’-TGCAGCTCATTCATGGCTCT-3’ |
| LXRβ | 5’-GATCTTTCTCCGACCAGCCC-3’ | 5’-CCACAATCTCCTGGACCGAG-3’ |
| FXR | 5’-TGACAAAGAAGCCGCGAATG-3’ | 5’-CACGGCGTTCTTGGTAATGC-3’ |
| PPARα | 5’-GCATTTGGGCGTATCTCACC-3’ | 5’-CTTGACCAGCCACAAACGTC-3’ |
| PPARγ | 5’-TGTCATCTACGACATGAGTTCCTT-3’ | 5’-GGGGGTGATATGTTTGAACTTG-3’ |
| PPARβ/δ | 5’-TATGCGCATGGGACTCACT-3’ | 5’-CCGTGGGTTTGTCTTCATCT-3’ |
| aP2 | 5’-CACCGCAGACGACAGGAAG-3’ | 5’-GCACCTGCACCAGGGC-3’ |
| CD36 | 5’-CTTGTGTTTTGAACATTTCTGCTT-3’ | 5’-TTGTACTATACTGTGCTAATGAGA-3’ |
| β-actin | 5’-CTTTGCAGCTCCTTCGTTGC-3’ | 5’-ACGATGGAGGGGAATACAGC-3’ |
| VDR | 5’- GAAGCGCAAGGCCCTGTT -3’ | 5’- CGCTGCACCTCCTCATCTGT -3’ |

**Table S2.** Primer sets used for plasmid construction and the VDRE promoter assay.

| VDRE candidate | Starting site | Forward primer | Reverse primer |
| --- | --- | --- | --- |
| 1 | -2200 | 5’-gtacctgagctcgctagcAGGTCTTGGGGTGAG-3’ | 5’-GATCTTGATATCCTCGAGCTCACCCCAAGACCT-3’ |
| 2 | -2043 | 5’-gtacctgagctcgctagcGCTTCATGGGGTTGT-3’ | 5’-GATCTTGATATCCTCGAGACAACCCCATGAAGC-3’ |
| 3 | -1867 | 5’-gtacctgagctcgctagcGCCTCAGAGGGTGCA-3’ | 5’-GATCTTGATATCCTCGAGTGCACCCTCTGAGGC-3’ |
| 4 | -1561 | 5’-gtacctgagctcgctagcGGGTCAGGAGGACA-3’ | 5’-GATCTTGATATCCTCGAGTGTCCTCCTGACCC-3’ |
| 5 | -634 | 5’-gtacctgagctcgctagcTCCAGTAGCTT-3’ | 5’-GATCTTGATATCCTCGAGGAAGCTACTGGA-3’ |
| 6 | -595 | 5’-gtacctgagctcgctagcGCTCCCTTGGCTC-3’ | 5’-GATCTTGATATCCTCGAGGAGCCAAGGGAGC-3’ |
| 7 | -80 | 5’-gtacctgagctcgctagcGTGCACTTGAAACA-3’ | 5’-GATCTTGATATCCTCGAGTGTTTCAAGTGCAC-3’ |
| 8 | -1 | 5’-gtacctgagctcgctagcGGTGCAGTGGCTCA-3’ | 5’-GATCTTGATATCCTCGAGTGAGCCACTGCACC-3’ |
| 9 | +162 | 5’-gtacctgagctcgctagcGAGGCAGGAGGATCA-3’ | 5’-GATCTTGATATCCTCGAGTGATCCTCCTGCCTC-3’ |
| 10 | +314 | 5’-gtacctgagctcgctagcGTGGCAGCAGGTCCA-3’ | 5’-GATCTTGATATCCTCGAGTGGACCTGCTGCCAC-3’ |

**Table S3. VD3 responsiveness of candidate VDRE upon transient transfection.**

| Mouse osteopontin VDRE | | 100% |
| --- | --- | --- |
| ① | -2.2±4.6 | |
| ② | -30.4±9.3 | |
| ③ | -29.8±7.8 | |
| ④ | -4.2±5.1 | |
| ⑤ | +10.5±10.9 | |
| ⑥ | -33.9±7.2 | |
| ⑦ | -41.5±5.5 | |
| ⑧ | -39.1±9.1 | |
| ⑨ | -21.8±1.8 | |
| ⑩ | -8.4±3.5 | |

Relative luciferase activity of each selected putative VDRE-luc-reporter construct compared by mouse osteopotin VDRE as the positive control. Each value was expressed as average ± SD derived from at least three experiments.
