# Supplementary figures and images for: Vitamin D3/VDR resists diet-induced obesity by modulating UCP3 expression in muscles
Source: J Biomed Sci. 2016 Jul 29;23:56. doi: 10.1186/s12929-016-0271-2 (PMC4966724; doi:10.1186/s12929-016-0271-2)

## Slide 1
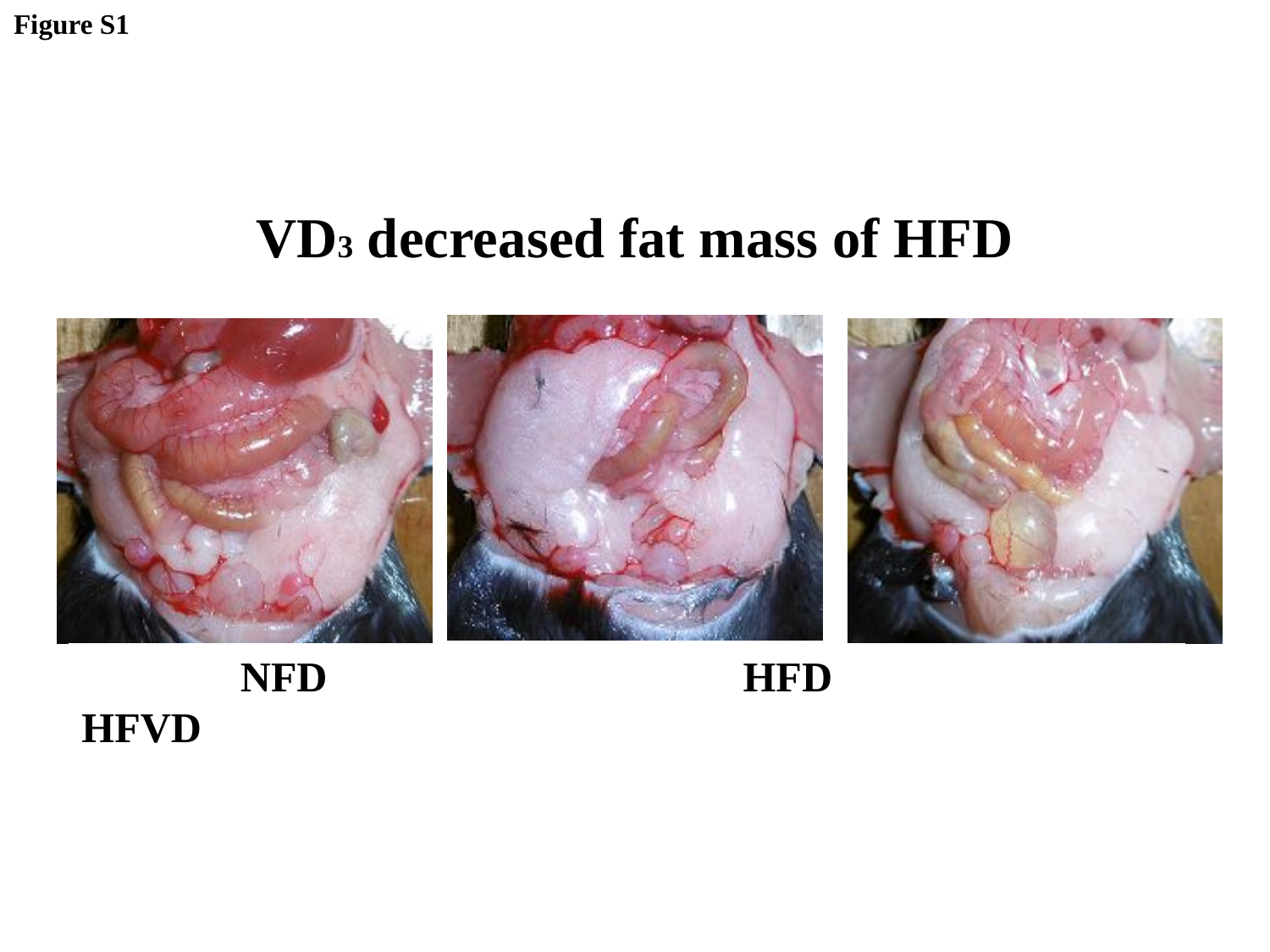

Figure S1
VD3 decreased fat mass of HFD
 NFD　　 　　　　　HFD HFVD

Supplement: Additional file 2: Figure S1. — Comparison of mesenteric fat tissue of representative mouse originated from the breeding stock of each group. Similar phenomena were noticed at the epididymis adipose tissues. (PPTX 281 kb) [file 12929_2016_271_MOESM2_ESM.pptx]

## Slide 1
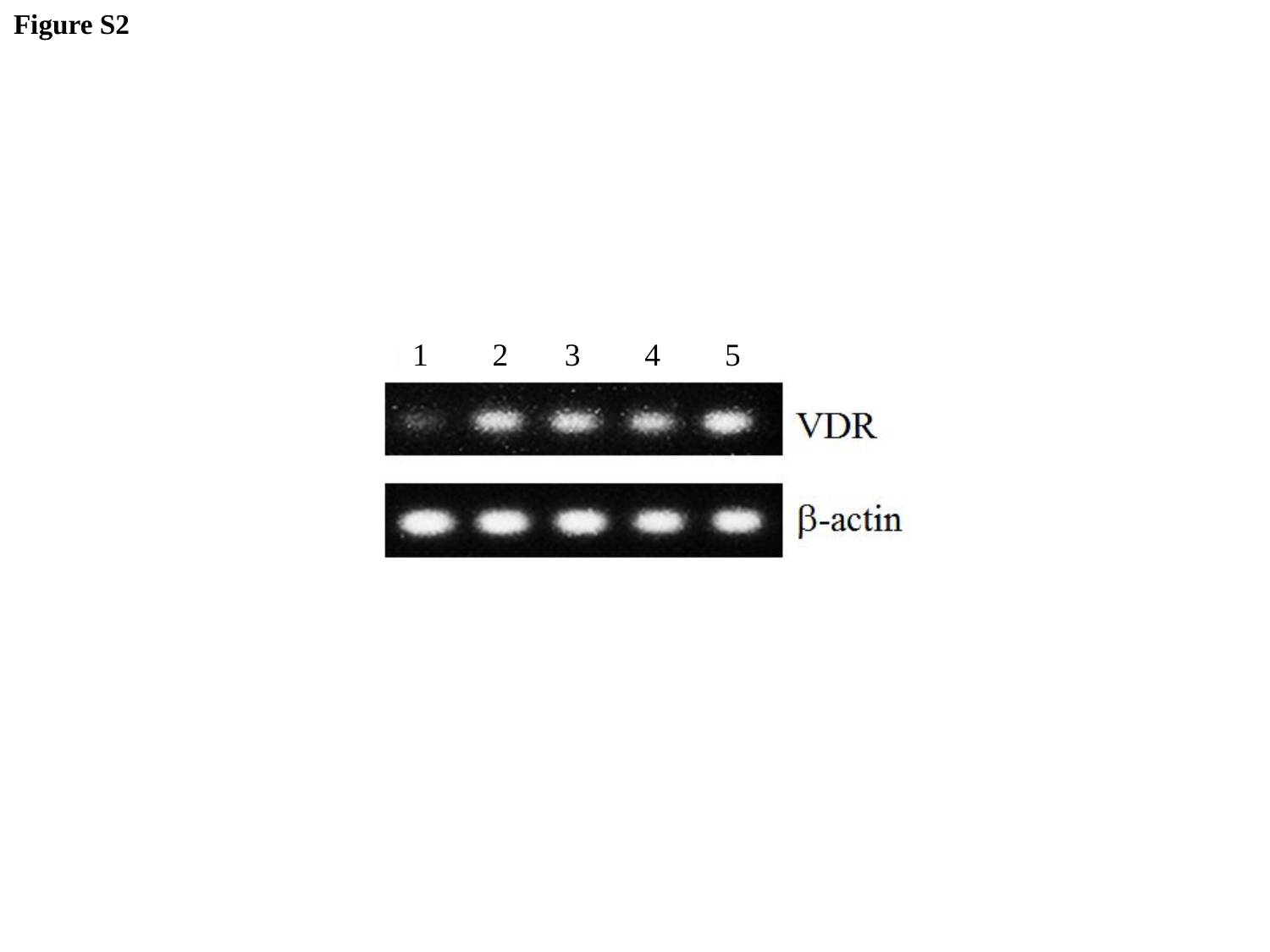

Figure S2
1 2 3 4 5

Supplement: Additional file 3: Figure S2. — VDR mRNA expression was detected in the (1) kidney adipose, (2) mesentery adipose, (3) epididymis adipose, (4) liver and (5) thigh rectus femoralis muscle via PCR gel electrophoresis. The sequences of the primer sets are shown in Additional file 1: Table S1. PCR products of the expected sizes were detected by agarose gel electrophoresis, suggesting that VDR transcripts were present in those tissues. (PPTX 88 kb) [file 12929_2016_271_MOESM3_ESM.pptx]

## Slide 1
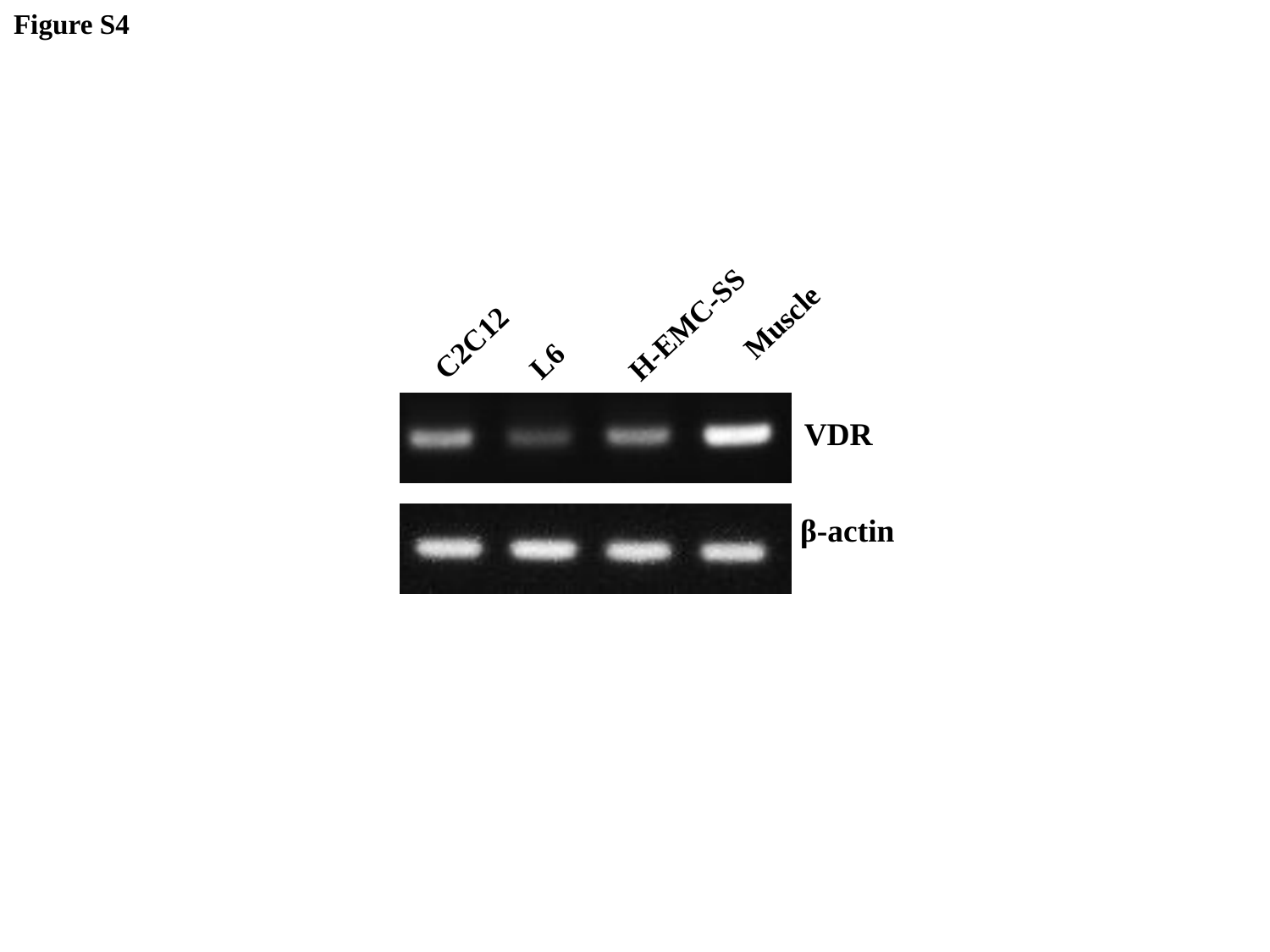

Figure S4
Muscle
H-EMC-SS
C2C12
L6
VDR
β-actin

Supplement: Additional file 5: Figure S4. — The presence of VDR transcripts was confirmed by RT-PCR analysis of C2C12 cells, L6 cells, H-EMC-SS cells and the rectus femoris muscle. PCR products of the expected sizes were detected by agarose gel electrophoresis, suggesting that the transcripts were present in each cell line. Beta actin was used as a positive control for PCRs. (PPTX 49 kb) [file 12929_2016_271_MOESM5_ESM.pptx]
