# Supplementary material for: Vitamin D3/VDR resists diet-induced obesity by modulating UCP3 expression in muscles
Source: J Biomed Sci. 2016 Jul 29;23:56. doi: 10.1186/s12929-016-0271-2 (PMC4966724; doi:10.1186/s12929-016-0271-2)
Supplement: Additional file 4: Figure S3. — UCP1 mRNA expression was detected in the abdominal and epididymal adipose via quantitative real time PCR. The sequences of the primer sets are shown in Additional file 1: Table S1. The expression level of UCP1 in the abdominal adipose tissues of HFDV were less than that of HFD. On the contrary, there was no significant difference between HFD and HFVD in expression level of UCP1 in the epididymal adipose tissue. UCP1 mRNA levels seem to be inconstant, depending on the relative content of browning cell number per volume of the fat. Although HFD treatment had modified the UCP1 mRNA expression levels even in the white adipose tissues, VD3 administration did not have any particular contributions to UCP1 expression to resist diet-induced obesity. (PPTX 47 kb) [file 12929_2016_271_MOESM4_ESM.pptx]

## Slide 1
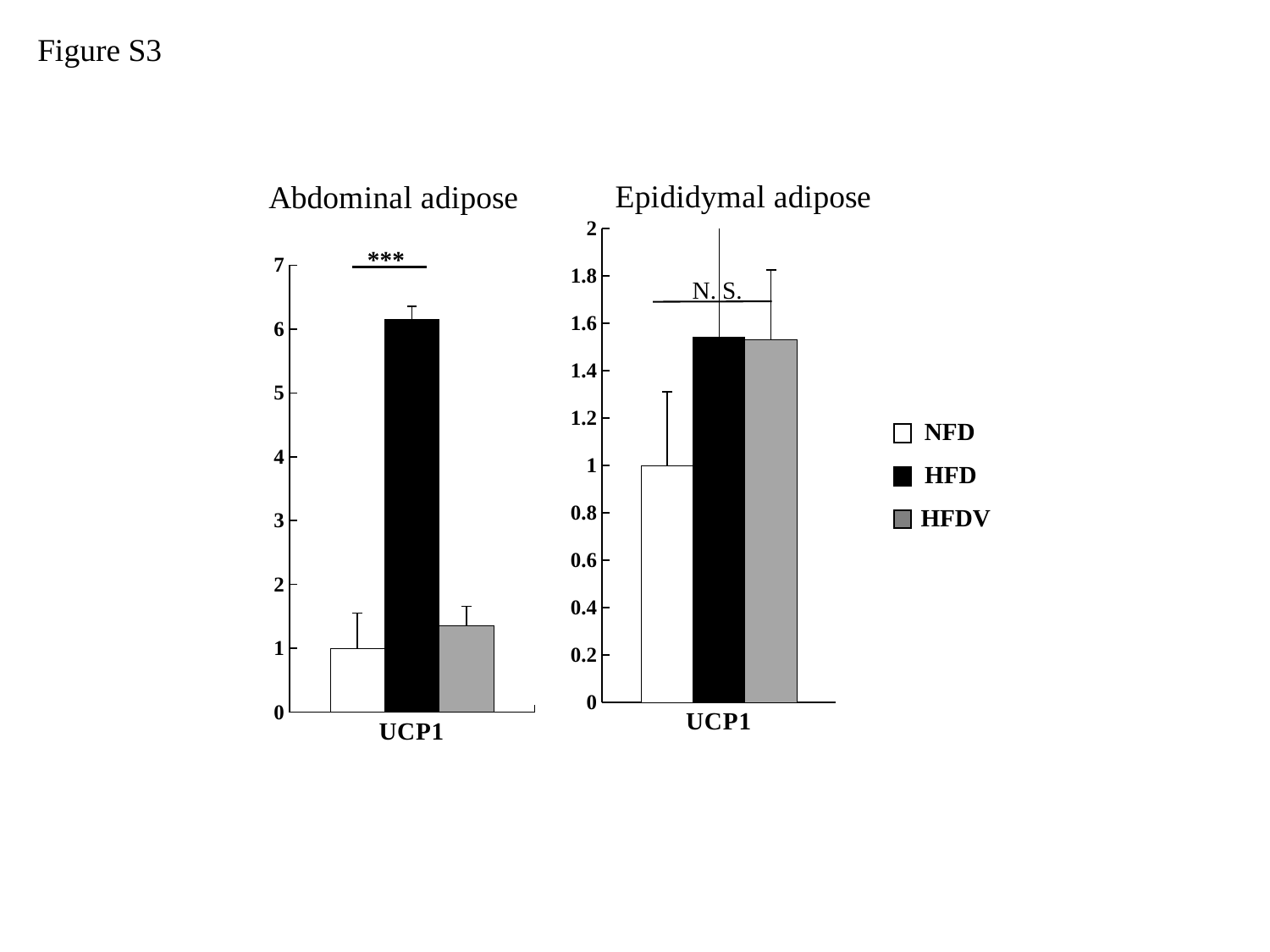

Figure S3
### Chart: Abdominal adipose
| Category | | | |
|---|---|---|---|
| UCP1 | 1.0 | 6.148788287589436 | 1.3476152588429084 |
| UCP2 | 1.0 | 0.9838487114304971 | 1.34487717162792 |
### Chart: Epididymal adipose
| Category | NFD | HFD | HFVD |
|---|---|---|---|
| UCP1 | 1.0 | 1.543593660982016 | 1.5314472183088363 |
| UCP2 | 1.0 | 1.1742902778114634 | 1.7599432034816926 |
***
N. S.
NFD
HFD
HFDV
